# Restoring Mitochondrial Quantity and Quality to Reverse Warburg Effect and Drive Tumor Differentiation

**DOI:** 10.21203/rs.3.rs-5494402/v1

**Published:** 2024-12-13

**Authors:** Jiangbin Ye, Haowen Jiang, Sarah Tiche, Clifford He, Junyan Liu, Fuyun Bian, Mohamed Jedoui, Balint Forgo, Md Tauhidul Islam, Meng Zhao, Pamela Emengo, Bo He, Yang Li, Albert Li, Anh Truong, Jestine Ho, Cathyrin Simmermaker, Yanan Yang, Meng-Ning Zhou, Zhen Hu, Katrin Svensson, Daniel Cuthbertson, Florette Hazard, Lei Xing, Hiroyuki Shimada, Bill Chiu

**Affiliations:** Stanford University; Stanford University; Stanford University; UC San Diego School of Medicine; Stanford University; Stanford University; Stanford University; Stanford University; Stanford University; Stanford University; Stanford University; Stanford University; Stanford University; Stanford University; Agilent Technologies; Agilent Technologies; Agilent Technologies; Agilent Technologies; Stanford University; Stanford University; Stanford University; Agilent Technologies; UC Davis; Stanford University; Stanford University; Stanford University

## Abstract

Reduced mitochondrial quality and quantity in tumors is associated with dedifferentiation and increased malignancy. However, it remains unclear how to restore mitochondrial quantity and quality in tumors, and whether mitochondrial restoration can drive tumor differentiation. Our study shows that restoring mitochondrial function using retinoic acid (RA) to boost mitochondrial biogenesis and a mitochondrial uncoupler to enhance respiration synergistically drives neuroblastoma differentiation and inhibits proliferation. U-^13^C-glucose/glutamine isotope tracing revealed a metabolic shift from the pentose phosphate pathway to oxidative phosphorylation, accelerating the TCA cycle and switching substrate preference from glutamine to glucose. These effects were reversed by ETC inhibitors or in ρ0 cells lacking mtDNA, emphasizing the necessity of mitochondrial function for differentiation. Dietary RA and uncoupler treatment promoted tumor differentiation in an orthotopic neuroblastoma xenograft model, evidenced by neuropil production and Schwann cell recruitment. Single-cell RNA sequencing analysis of the orthotopic xenografts revealed that this strategy effectively eliminated the stem cell population, promoted differentiation, and increased mitochondrial gene signatures along the differentiation trajectory, which could potentially significantly improve patient outcomes. Collectively, our findings establish a mitochondria-centric therapeutic strategy for inducing tumor differentiation, suggesting that maintaining/driving differentiation in tumor requires not only ATP production but also continuous ATP consumption and sustained ETC activity.

## Introduction

The Warburg effect, the metabolic hallmark of cancer, is prevalent in cancers and underpins the principle of positron emission tomography–computed tomography (PET-CT) imaging, characterized by a disproportionately elevated glycolytic flux compared to the tricarboxylic acid (TCA) cycle even under aerobic conditions ^1,2^. Although the Warburg effect appears to occur in the cytosol, its underlying cause is mitochondrial impairment ^3,4^.

The mitochondrial impairment in cancers can result from reduced mitochondrial quantity and/or quality. Many cancers exhibit lower mitochondrial DNA (mtDNA) copy numbers, and decreased levels of mitochondrial RNA and respiratory proteins encoded by nuclear DNA compared to their corresponding healthy tissues, all of which suggest a reduction in mitochondrial quantity within these tumors ^5,6^. In addition, slow TCA cycle flux and low ATP synthesis rate was observed in cancers compared to their corresponding healthy tissues ^7,8^, regardless of whether mtDNA copy numbers are reduced or unchanged, indicating the impaired mitochondrial quality in tumors. Additionally, compared to normal cells, the mitochondria in cancer cells exhibit higher levels of fission, lower levels of fusion, and disrupted quality control processes, resulting in mitochondrial fragmentation and enhanced aerobic glycolysis ^9^.

It may seem counterintuitive that mitochondrial impairment not only fails to impede but actually leads to cancer progression, even though cancer cells continue to generate most of their ATP through the mitochondrial TCA cycle ^7,8^. Although mitochondrial impairment, particularly through electron transport chain (ETC) inhibition, reduces proliferation by disrupting aspartate and pyrimidine synthesis ^10–12^, it also significantly drives cells dedifferentiation in tumor cells ^13–15^. This dedifferentiation process initially reduces ATP demand by shedding energy-intensive tissue-specific functions, and ultimately leads to malignancy and a poorer prognosis. Mechanistically, mitochondrial impairment drives this malignant progression by disrupting NAD^+^ regeneration through respiration, leading to a lower NAD^+^/NADH ratio compared to non-cancerous cells ^16^. This reduced NAD^+^/NADH ratio not only drives the conversion of pyruvate to lactate, but also many other metabolic reprogramming events, such as reductive carboxylation^17–20^ and L-2HG production^21–23^, which inhibit normal differentiation while promoting tumor progression ^24^. Furthermore, an impaired TCA cycle reallocates metabolites from energy production to biosynthetic processes, actively facilitating uncontrolled malignant cell proliferation ^1^.

Differentiated cells generally consume more ATP than stem or progenitor cells, suggesting that the capacity to generate sufficient ATP has been essential for enabling cellular differentiation throughout evolution. But in modern multicellular organisms, maintaining this differentiated state requires not only ATP production, but also its continuous consumption to sustain ETC activity, which drives the forward TCA cycle, producing intermediates essential for epigenetic modifications ^25^. These modifications, in turn, play a crucial role in maintaining the differentiated state by regulating gene expression patterns that define and stabilize cell identity ^26^. In this context, our study aims to address the critical question of whether restoring mitochondrial quantity and quality can drive tumor redifferentiation and to explore the methods for achieving this.

In our research, we utilize neuroblastoma as a model. Neuroblastoma accounts for 15% of pediatric cancer-related fatalities, and patient survival strongly correlates with its differentiation status, with better differentiation predicting improved outcomes ^27–29^. Therefore, inducing differentiation in these tumors may significantly enhance survival and quality of life for affected children. Retinoic acid (RA), a derivative of vitamin A, is known to downregulate N-Myc and induce neuroblastoma differentiation ^30–34^, making it an FDA-approved therapy for increasing event-free survival in neuroblastoma patients ^35^. However, some patients develop resistance to RA and experience relapse, indicating that RA alone is insufficient; combining it with other treatments may be necessary to achieve more effective differentiation. We demonstrated that RA treatment increased mtDNA copy number and promoted mitochondrial elongation in neuroblastoma cells; however, this upregulation of mitochondrial biogenesis did not lead to enhanced mitochondrial respiration. To overcome this limitation, we employed a mitochondrial uncoupler to dissipate the mitochondrial membrane potential, thereby activating mitochondrial respiration and the ETC, increasing the NAD^+^/NADH ratio, reversing the Warburg effect, and enhancing mitochondrial quality. Using this strategy, we were able to restore the mitochondrial quantity and quality, prompting a metabolic switch from biosynthesis to energetics in cancer cells. This metabolic reprogramming induced differentiation both *in vitro* and *in vivo*. Functional or genetic inhibition of mitochondria respiration in cancer cells abolished the differentiation effect of RA and uncoupler, indicating that mitochondrial respiration is essential for tumor cell differentiation. Single-cell RNA sequencing analysis further revealed changes in cell identity following RA and uncoupler intervention. Histological evidence and patient gene signature prognosis analysis suggest that this mitochondria restoration strategy could significantly improve patient outcomes. Highlighting the pivotal role of mitochondrial homeostasis in cancer cell differentiation status, our research introduces a novel therapeutic approach to efficiently restore mitochondrial quantity and quality, centered on the differentiation of tumors, and offers a promising novel avenue for cancer treatment.

## Results

### Retinoic Acid Increases Mitochondrial Quantity without Activating the TCA Cycle

**Figure 1:**
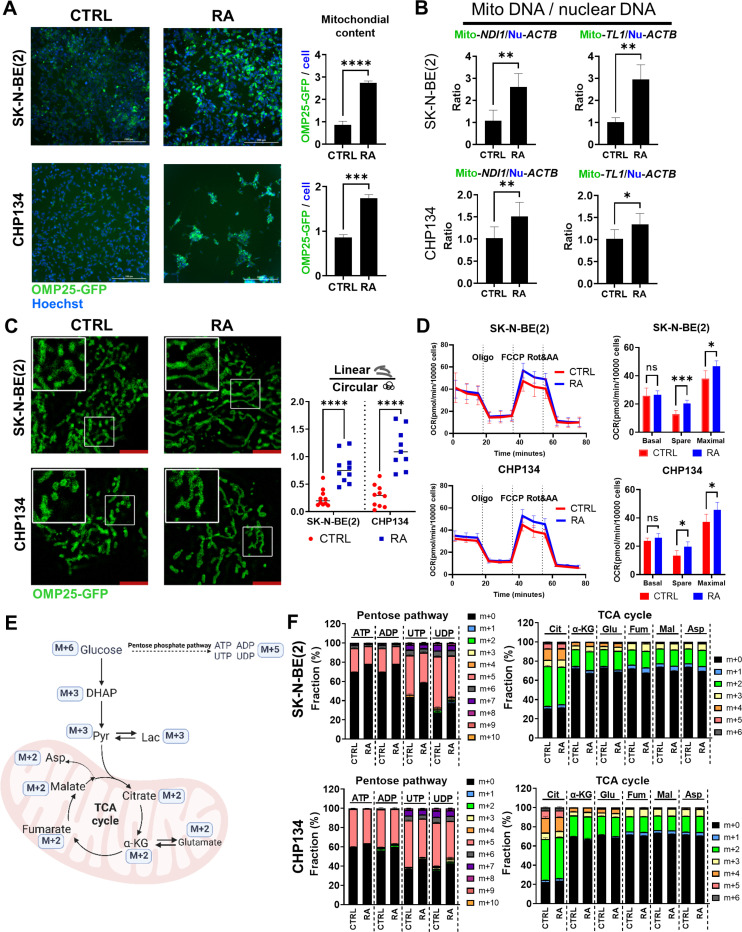
RA increased mitochondrial quantity without activating mitochondrial respiration (A) SK-N-BE(2) and CHP134 cells stably expressing 3XHA-OMP25-GFP (green) were treated with DMSO (CTRL) or 1 μM RA for 72 hours. Hoechst dye (blue) was used to stain nuclei, and the GFP signal was normalized to the number of Hoechst-stained nuclei. (B) The mitochondrial DNA (mtDNA) to nuclear DNA ratio was quantified using qPCR by analyzing the mitochondrial genes *ND1* and *TL1*, normalized to the nuclear gene *ACTB*. (C) Mitochondrial morphology was visualized via confocal microscopy with a 63x oil lens and 2x zoom, with linear and circular mitochondria analyzed using ZEN 3.9 software. (D)Mitochondrial function was assessed through Seahorse assays, measuring basal, spare, and maximal OCR. (E) A schematic of U-^13^C-glucose tracing. (F) Glucose labeling fractions in the pentose phosphate pathway and TCA cycle metabolites were measured by LC-MS.

Retinoic acid (RA), a derivative of vitamin A, has been used clinically to induce tumor cell differentiation, particularly in acute promyelocytic leukemia (APL) and neuroblastoma ^35–37^. While previous studies have attributed the differentiation effect of RA to its role as a ligand for RA receptors, transcription factors that activate nuclear transcription. By reanalyzing the RA target genes, we demonstrated that RA significantly upregulated nuclear-encode mitochondrial genes, particularly those involved in the mitochondrial ribosome and complex I subunits (Fig S1A), which are largely ignored in the previous studies. This intriguing discovery leads us to hypothesize that RA induces differentiation in part by modulating mitochondrial function. Given the growing evidence that mitochondrial metabolism plays a key role in epigenetic regulation—a critical driver of cellular differentiation—RA’s effects on differentiation may partly arise from its influence on mitochondrial function, in addition to its known role in activating nuclear receptors ^15,38–40^. To study this, we employed a 3xHA-OMP25-GFP reporter system ^41^ to monitor mitochondrial dynamics in neuroblastoma cells during differentiation (Figure S1B). This system enabled real-time observation of mitochondrial mass changes upon RA-induced differentiation (Supplemental Video 1). Retinoic acid (RA) treatment significantly increased mitochondrial mass, as evidenced by increased GFP intensity and elevated mtDNA copy number (Fig. 1A, 1B, S1C). The latter was determined by the ratio of mitochondrial DNA (mtDNA) to genomic DNA (gDNA), using mitochondrial-encode genes *MT-ND1* (mitochondrially encoded NADH dehydrogenase 1) and *MT-TL1* (mitochondrially encoded tRNA leucine 1), along with the nuclear-encode genes *ACTB* and *B2M* (β2 microglobulin) (Figure S1C) (p < 0.05). Additionally, RA treatment significantly altered mitochondrial morphology, increasing the proportion of linear mitochondria compared to circular ones (Fig. 1C) (p < 0.05), indicating an increase in mitochondrial fusion events.

Intriguingly, despite the increased mitochondrial content and changes in morphology, RA treatment did not enhance the basal respiration rate (Fig. 1D). This indicates an unchanged baseline energy demand and suggests decreased respiration activity per mitochondrion. However, both the spare respiratory capacity and maximal respiration capacity were increased (Fig. 1D), suggesting that while the resting metabolic rate remains unchanged, the mitochondria are functional and can be activated for respiration. To further elucidate the metabolic reprogramming effects of RA treatment, we performed U- C-glucose tracing experiments. The results revealed a slight decrease in glucose entry into the pentose phosphate pathway following RA treatment, as indicated by the m + 5 fraction of ATP, ADP, UTP and UDP, with neglectable changes in the glycolysis pathway and TCA cycle (Fig. 1E, 1F and S1D). This is consistent with the Seahorse data (Fig. 1D), confirming that although RA treatment increased mitochondrial quantity, overall respiration was not enhanced.

### Mitochondrial Uncoupler Activates Mitochondrial Respiration, Redirecting Metabolism from Biosynthesis to Energetics

**Figure 2:**
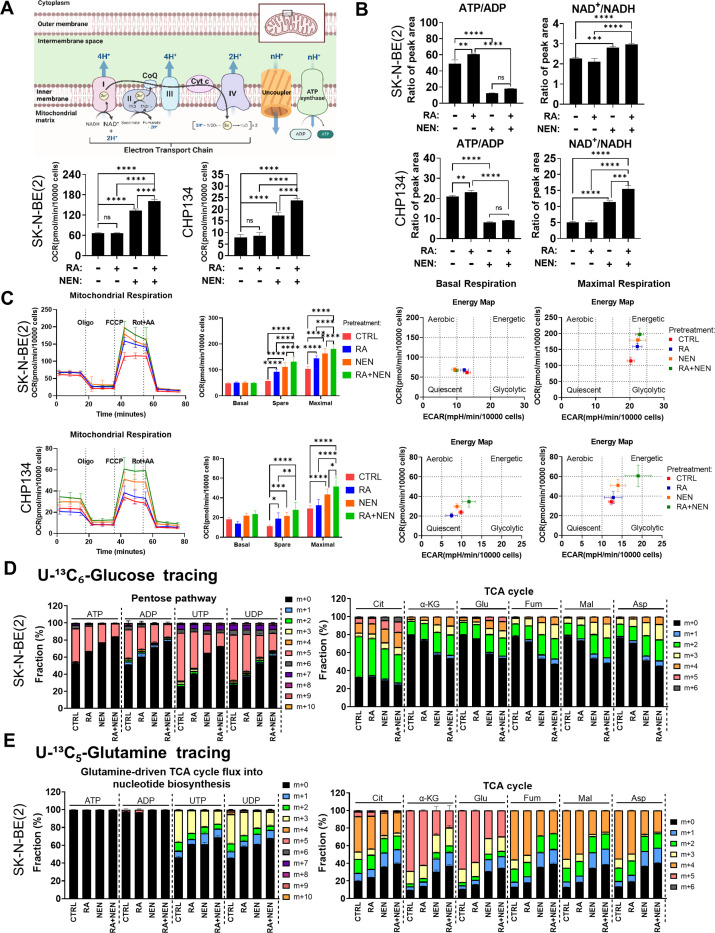
Mitochondrial uncoupler synergizes with RA to activate mitochondrial respiration and shift metabolism from anabolism to catabolism. (A) Top panel: Schematic of the oxidative phosphorylation illustrating how uncouplers reduce proton gradients, thereby stimulating mitochondrial respiration. Bottom panel: Basal oxygen consumption rates (OCR) were measured using the Seahorse analyzer in SK-N-BE(2) and CHP134 cells following a 72-hour treatment with 1 μM RA, 1 μM NEN, RA+NEN, or vehicle. (B) ATP/ADP and NAD^+^/NADH ratios in SK-N-BE(2) and CHP134 cells treated with RA, NEN, or a combination, measured by LC-MS. (C) Left: Mitochondrial respiration measured via Seahorse assays shows basal, spare, and maximal respiration in SK-N-BE(2) and CHP134 cells pre-treated with CTRL, RA, NEN, or RA+NEN. Right: Energy maps display the shifts in mitochondrial respiration and glycolytic activity under different treatment conditions. (D) U-^13^C_6_-glucose and (E) U-^13^C_5_-glutamine tracing, measured by LC-MS, illustrate the labeling fraction changes of the pentose phosphate pathway and TCA cycle metabolites upon CTRL, RA, NEN, or RA+NEN treatment conditions.

We hypothesized that RA is unable to activate mitochondrial respiration in cancer cells due to the absence of a robust ATP consumption mechanism. As a result, ATP accumulates, preventing ETC respiration from activating, despite an increase in mitochondrial number (Fig. 2B). To overcome this limitation, we employed the previously tested mitochondrial uncoupler, niclosamide ethanolamine (NEN) ^23^, to create a pseudo-ATP demand by dissipating the mitochondrial membrane potential, thereby increasing respiration and lowering ATP synthesis efficiency simultaneously. As expected, NEN treatment significantly increased cellular basal respiration, and the combination of RA and NEN further enhanced this effect (Fig. 2A). Meanwhile, NEN treatment reduced the intracellular ATP/ADP ratio, thereby activating the ETC and consequently increasing the intracellular NAD^+^/NADH ratio (Fig. 2B). To evaluate the long-term effects of RA and the uncoupler on enhancing mitochondrial respiration, cells were pretreated with either RA, NEN, RA + NEN, or no treatment for 72 hours, then replated without the uncoupler or RA for Seahorse analysis to measure respiration capacity (Figure S2A). Surprisingly, spare respiratory capacity and maximal respiratory capacity were significantly increased by NEN pretreatments, with the RA + NEN combination treatment showing further enhancement compared to NEN alone (Fig. 2C, left). Energetic mapping also revealed that RA + NEN treatment shifted the cells to a more energetic state (Fig. 2C, right). These findings suggest that the uncoupler does not only acutely increase oxygen consumption, but also further enhances the maximal oxygen consumption capacity with prolonged effect.

To elucidate the metabolic reprogramming effects of RA + uncoupler combination, U-C-glucose/glutamine isotope tracing was performed. The results showed that NEN treatments significantly reduced glucose carbon into the pentose phosphate pathway and nucleotide synthesis, as indicated by reduced m + 5 ATP, ADP, UTP and UDP, and reduced glutamine-driven aspartate into pyrimidine biosynthesis, as indicated by reduced m + 3 UTP and UDP (Fig. 2D and Figure S2B,D). The combination of RA + NEN further reduced both glucose carbon into the pentose phosphate pathway and glutamine-driven carbon into pyrimidine biosynthesis. The treatments had no significant effect on the glycolytic pathway, as indicated by unchanged m + 3 fraction of dihydroxyacetone phosphate (DHAP), 3-phosphoglycerate (3PG), phosphoenolpyruvate (PEP), pyruvate, and lactate (Figure S2C-D). However, NEN treatment significantly increased glucose flux into the TCA cycle and accelerated the TCA cycle, as determined by the sustained increased fractions of m + 2 (2nd cycle) and m + 3/4 (2nd cycle) in citrate, α-ketoglutarate, glutamate, fumarate, malate, and aspartate, m + 5 (3rd cycle) in glutamate, and m + 6 (4th cycle) in citrate (Fig. 2D,and Figure S2D). The combination of RA + NEN showed the greatest increase in these labeling fractions. Meanwhile, glutamine tracing also supported the observation of TCA cycle acceleration, as determined by increased fractions of m + 3 (2nd cycle) and m + 1/2 (3rd cycle) in α-ketoglutarate, and m + 1 (3rd cycle) in fumarate, malate, and aspartate (Fig. 2E, S2E). NEN treatment inhibited reductive carboxylation, as indicated by reduced m + 3 fumarate, malate, and aspartate (Fig. 2E and Figure S2E), consistent with our previous publication ^20^, and RA + NEN combination further reduced the labeling fraction of reductive carboxylation products m + 3 malate and m + 3 aspartate (Figure S2E). Together, the tracing results demonstrated that RA + NEN combination shifts the TCA cycle’s substrate preference from glutamine to glucose and redirects metabolism from biosynthesis to energetics.

### Restoring Mitochondrial Quantity and Quality Drives Differentiation

**Figure 3:**
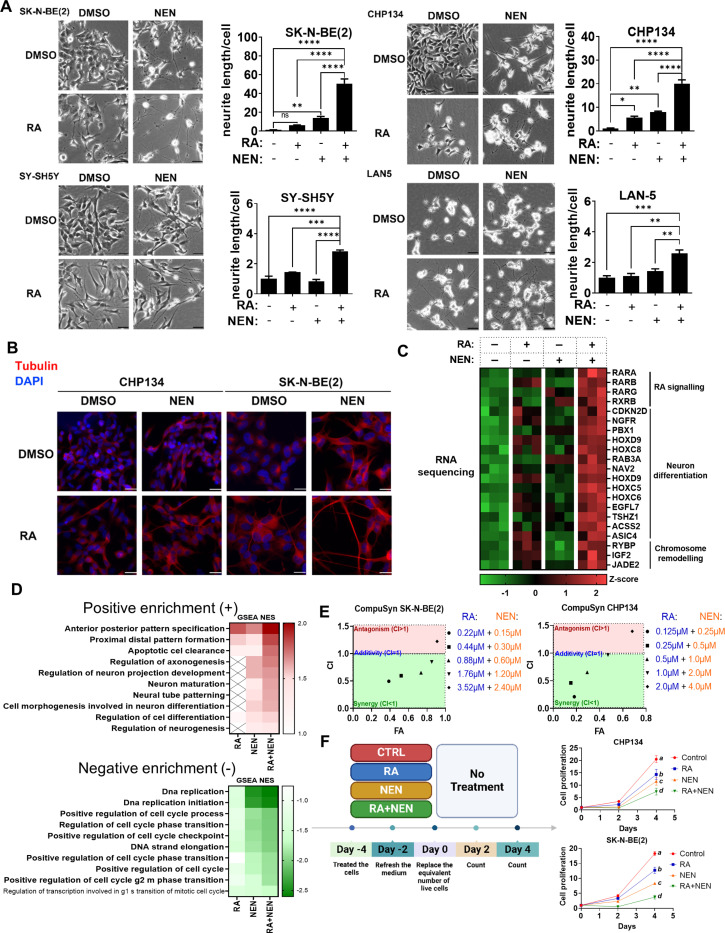
RA and mitochondrial uncoupler synergize to induce neuronal differentiation in neuroblastoma cells. (A) Phase-contrast images of SK-N-BE(2), CHP134, SY-SH5Y, and LAN-5 neuroblastoma cells treated with DMSO (CTRL), RA, NEN, or a combination of RA+NEN. Neurite outgrowth was quantified by measuring the average neurite length per cell. Bar graphs depict neurite length for each condition. Scale bar: 25μM. (B) Immunofluorescence staining of neuron marker β-Tubulin-III (red) and DAPI (blue) in CHP134 and SK-N-BE(2) cells treated with DMSO, RA, or NEN, showing enhanced neurite formation upon treatment. (C) Heatmap showing differential expression of genes related to RA signaling, neuron differentiation, and chromosome remodeling in SK-N-BE(2) cells treated with RA, NEN, or a combination. (D) Gene set enrichment analysis (GSEA) illustrating positive and negative enrichment of biological pathways in cells treated with RA, NEN, or RA+NEN. E) Chou-Talalay analysis of SK-N-BE(2) and CHP134 cells treated with varying concentrations of RA and NEN, showing synergistic effects in inhibiting cell proliferation (CI values were calculated using CompuSyn). (F) Left panel: Experimental timeline for cell proliferation assays. Cells were pre-treated with CTRL, RA, NEN, or RA+NEN, and cell counts were measured every two days in the absence of treatment. Right panel: Growth curves illustrate cell proliferation under each conditions for SK-N-BE(2) and CHP134 cells.

Differentiation is associated with mitochondrial biogenesis and enhanced respiration ^15,40^. Therefore, we asked whether the combination of RA + uncoupler could activate differentiation in cancer cells. RA or NEN alone initiated the differentiation in neuroblastoma cell lines SK-N-BE(2), CHP134, SH-SY5Y and LAN5, as determined by the morphology changes and total neurite length per cell measure, with the RA + NEN group showing a synergistic enhancement in differentiation (Fig. 3A). Notably, the cause of increased total neurite length per cell was mainly due to a synergistic increase in neurite counts per cell, not average neurite length increase (Figure S3A). Immunostaining for neuron differentiation marker β-Tubulin III confirmed the successful differentiation of treated cells into neurons, as evidenced by the high expression levels and distinct neuronal morphology compared to untreated cells (Fig. 3B). Other uncouplers, such as FCCP and BAM15 ^42^, were also tested in combination with RA and showed similar synergistic effects observed with RA + NEN, confirming that the differentiation depends on mitochondrial uncoupling (Figure S3B). RNA sequencing results showed a synergistic induction of genes associated with RA signaling (*RARB*, *RARG*), neuronal differentiation (*HOXC5*, *PBX1*), and chromatin remodeling (*RYBP*, *JADE2*), which are positively correlated with favorable prognosis in neuroblastoma (Fig. 3C, S3C). The gene expression changes observed in the RNA sequencing results were further validated by RT-PCR in a time-dependent manner (Figure S3D). Further analysis of gene set enrichment (GSEA) revealed positive enrichment of pathways related to neuronal differentiation and development, including anterior-posterior pattern specification, regulation of axonogenesis, neuron maturation, and regulation of neurogenesis in the RA + NEN group (Fig. 3D). Additionally, negative enrichment was observed in pathways related to cell cycle progression and DNA replication, such as DNA replication initiation, and positive regulation of the cell cycle phase transition (Fig. 3D). These findings indicate that the combination treatment not only promotes neuronal differentiation, but also suppresses proliferative pathways, aligning with the fact that proliferation arrest is usually associated with terminal differentiation.

To functionally validate the synergy between RA and NEN, we employed the Chou-Talalay method to calculate combination index (CI) ^43^, using cell proliferation as the readout on two neuroblastoma cell lines, SK-N-BE(2) and CHP134. The results demonstrated a strong synergistic effect of the combined RA and NEN treatment across different concentrations until the effect was saturated at higher concentrations, confirming that the combination therapy is more effective in inhibiting proliferation than either treatment alone (Fig. 3E). To assess the lasting effect on cell proliferation inhibition, cells were pretreated with either RA, NEN, RA + NEN, or left untreated for three days. They were then replated at equal numbers of live cells without further treatment, and their proliferation rates were monitored over time (Fig. 3F). The results showed that while RA and NEN pre-treatment individually reduced cell proliferation compared to the control, the combination of RA + NEN resulted in a significantly greater reduction in proliferation in both SK-N-BE(2) and CHP134 cell lines. This suggests that RA and NEN reduce proliferation not through a temporary effect on blocking the cell cycle, but more likely through a lasting effect on inducing differentiation.

### Pharmacological or Genetic Inactivation of Mitochondria Blocks Differentiation

**Figure 4:**
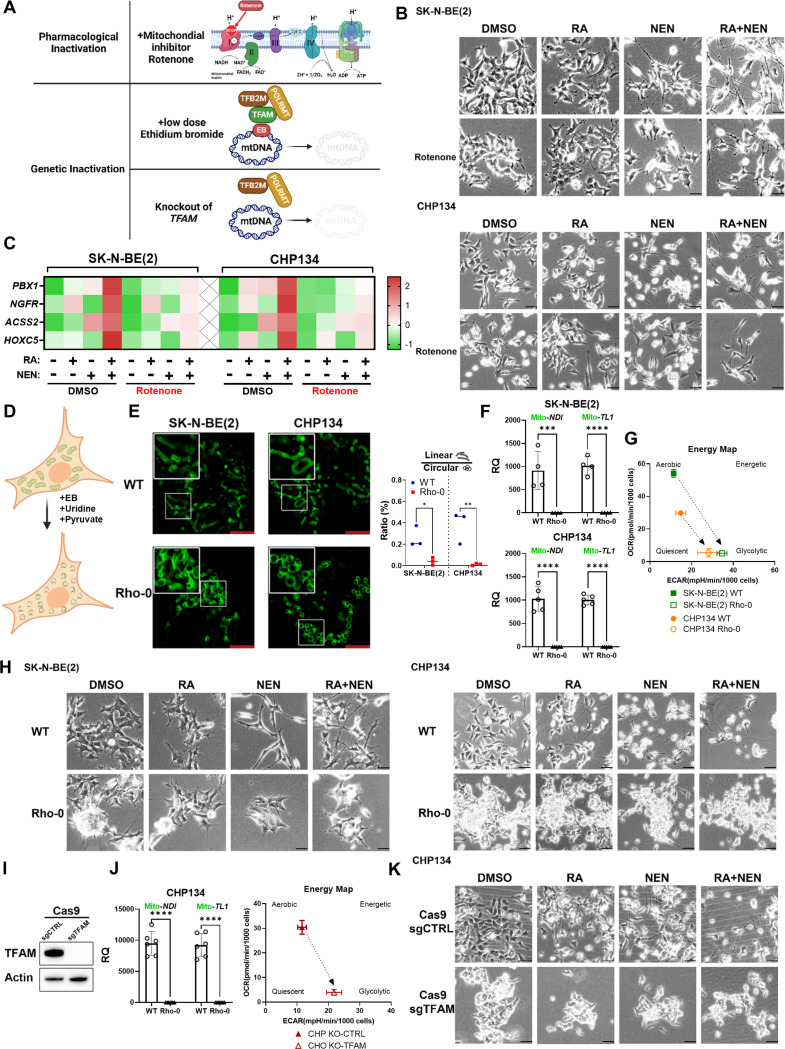
Mitochondrial respiration is essential for neuroblastoma differentiation. (**A**) Schematic illustrating three methods of mitochondrial inactivation. Pharmacological inactivation is achieved by Rotenone, a complex I inhibitor that disrupts the electron transport chain. Genetic inactivation includes low-dose ethidium bromide (EB), which inhibits mitochondrial DNA (mtDNA) replication by interfering with TFAM, TFB2M, and POLRMT, and TFAM knockout, which leads to complete mtDNA depletion, impairing mitochondrial function. (B) Phase-contrast images of SK-N-BE(2) and CHP134 cells treated with DMSO, RA, NEN, RA+NEN, with or without rotenone. (C) Heatmap displaying gene expression related to RA signaling and differentiation in SK-N-BE(2) and CHP134 cells under different treatments, measured by q-RT-PCR. (D) Schematic of ρ0 cell generation through EtBr treatment. (E) Left: Confocal images showing mitochondrial morphology in WT and ρ0 cells. Right: the ratio of linear/circular mitochondria. (F) mtDNA quantification in SK-N-BE(2) and CHP134 WT and ρ0 cells. (G) Energy map of mitochondrial respiration in WT and ρ0 cells. (H) Phase-contrast images of WT and ρ0 cells treated with DMSO, RA, NEN, or RA+NEN. (I) Western blot of TFAM and β-actin in CHP134 cells with TFAM knockout. (J) mtDNA quantification and energy map for CHP134 sgCTRL and sgTFAM cells. (K) Phase-contrast images of CHP134 sgCTRL and sgTFAM cells treated with DMSO, RA, NEN, or RA+NEN.

To determine whether mitochondrial respiration is essential for neuroblastoma differentiation, we assessed the impact of mitochondrial dysfunction on RA and NEN-induced differentiation. Neuroblastoma cells were treated with RA, NEN, or RA + NEN in the presence of mitochondrial complex I inhibitor rotenone (Fig. 4A). The results showed that complex I inhibition significantly blocked differentiation in neuroblastoma cells, as indicated by decreased total neurite length per cell, fewer neurite counts, and reduced expression of neuron differentiation-related genes compared to treatments without mitochondrial inhibitors (Fig. 4B, 4C, S4A). This suggests that mitochondrial respiration is essential for neuronal differentiation induced by RA and NEN.

Additionally, we employed a prolonged ethidium bromide (EB) treatment protocol to generate rho0 cells ^44^, which lack mitochondrial DNA and thus have severely compromised mitochondrial respiration (Fig. 4D). We also examined the mitochondrial morphology using the 3xHA-OMP25-GFP reporter system. Interestingly, the mitochondria lost their linear morphology and formed a hollow cycle (Fig. 4E). The mtDNA measurement further validated that the rho0 cells had almost undetectable mtDNA content compared to wild-type cells (Fig. 4F). Seahorse analysis further confirmed diminished mitochondrial respiration, as indicated by low oxygen consumption rates (OCR) and no response to uncoupler treatment, along with increased extracellular acidification rates (ECAR), a sign of the Warburg effect (Fig. 4G, S4B). Notably, the rho0 cells formed multilayer colonies, a characteristic of cells that have lost contact inhibition, suggesting increased malignancy (Fig. 4H). These rho0 cells treated with RA, NEN, or RA + NEN showed markedly reduced differentiation compared to WT cells, with significantly shorter neurite lengths and fewer neurites per cell (Fig. 4H, S4C). To address potential off-target effects of EB treatment that could inhibit cell differentiation, we knocked out mitochondrial transcription factor A (TFAM), a key activator of mitochondrial transcription and DNA replication (Fig. 4I). TFAM knockout depletes mitochondrial DNA and abolished respiration (Fig. 4J, S4D). Like rho0 cells, TFAM-knockout cells displayed resistance to RA and NEN-induced differentiation (Fig. 4K, S4E). These findings underscore the importance of intact mitochondrial for the differentiation of neuroblastoma cells induced by RA and NEN, highlighting the crucial role of mitochondrial homeostasis in supporting effective differentiation.

### Dietary Retinoic Acid and Mitochondrial Uncoupler Induce Tumor Differentiation in Orthotopic Neuroblastoma Xenografts

**Figure 5:**
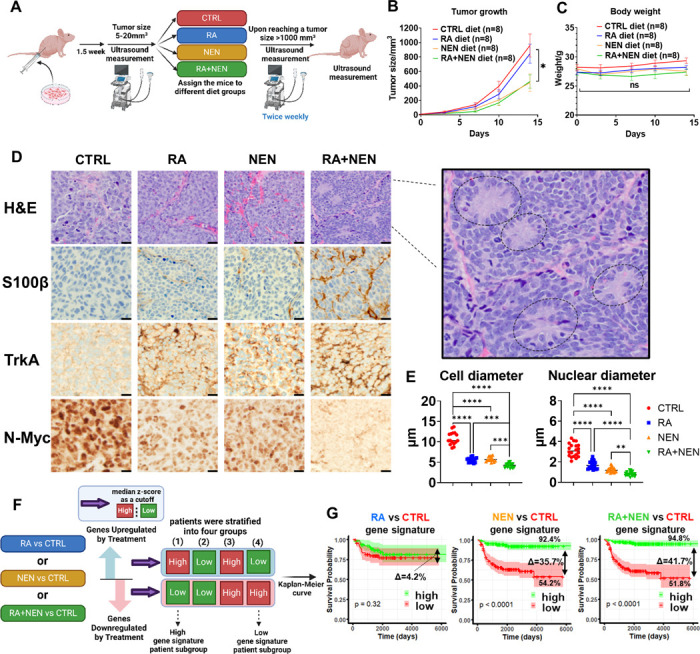
Effects of RA and NEN treatment on tumor differentiation in an orthotopic neuroblastoma model and potential beneficial impact on patient survival. (A) Schematic representation of the experimental design showing ultrasound monitoring, dietary interventions (CTRL, RA, NEN, and RA+NEN), and tumor size measurements until euthanasia. (B) Tumor growth curves displaying the effects of different diets on tumor volume over time. (C) Body weight measurements of mice under various diet conditions to assess treatment tolerance. (D) Representative histological sections with H&E staining and immunohistochemistry (IHC) for S100β, TrkA, N-Myc, and NF across treatment groups. (E) Quantification of cell and nuclear diameters, as well as the mitosis-karyorrhexis index (MKI), under different treatments. (F) Schematic illustrating bioinformatic analysis to stratify patient subgroups based on gene signature upregulation and downregulation. (G) Kaplan-Meier survival curves for patients with low or high RA, NEN, and RA+NEN treatments gene signatures.

Next, we investigated whether the combination of RA and a mitochondrial uncoupler could induce tumor differentiation *in vivo*. To assess this, we administered diets containing either 83.3 ppm RA, 2000 ppm NEN, or a combination of both (Fig. 5A). As we previously reported ^45^, NEN treatment significantly slowed tumor growth (Fig. 5B), while the combination of RA + NEN did not further reduce tumor size. None of these treatments affected the weight of the mice, indicating good safety and tolerance (Fig. 5C). Importantly, H&E staining revealed signs of tumor differentiation, such as neuropil production and rosette formation (Fig. 5D). Interestingly, the expression of the Schwann cell marker S100β and the neuronal differentiation marker TrkA—both indicators of favorable prognosis—was significantly elevated in both the RA and NEN groups, with the most pronounced effect observed in the combination group (Fig. 5D, S5A). Along with inducing differentiation, RA or NEN treatment reduced tumor cell size and nuclear size, with the combination treatment showing the greatest reduction—all indicators of improved prognosis (Fig. 5D, E). Conversely, N-Myc, a differentiation inhibitor ^31,32,46^ and major unfavorable prognosis marker in neuroblastoma, was reduced by either RA or NEN, with the combination group showing the most significant reduction (Fig. 5D, S5A).

To further evaluate the potential therapeutic effects of RA and NEN, a bioinformatic analysis of patient datasets was conducted, focusing on the transcriptional changes induced by RA, NEN, or their combination, and their correlation with clinical outcomes. The average z-scores for both upregulated and downregulated gene lists were calculated using the published neuroblastoma patient data (GSE62564). Using the median Z-score as a cutoff, patients were first separated into high and low gene signature expression groups. Based on this, we then stratified patients into four categories by considering both upregulated (high/low) and downregulated (high/low) gene expression levels: (1) high upregulated and low downregulated, (2) low upregulated and low downregulated, (3) high upregulated and high downregulated, and (4) low upregulated and high downregulated (Fig. 5F).These groups were then used to generate survival curves (Figure S5B). Notably, groups (1) and (4) represent the two extremes of the treatment gene signatures, categorized as ‘High’ and ‘Low’ treatment gene signatures, respectively. Interestingly, the RA-induced ‘High’ and ‘Low’ gene signatures did not significantly separate the survival curves, aligning with the observation that RA alone does not improve overall survival in neuroblastoma ^47–49^. However, the NEN-induced ‘High’ and ‘Low’ gene signatures significantly separated the survival curves, with the ‘High’ gene signature group showing a 92.4% overall survival rate, compared to 54.2% in the ‘Low’ gene signature group, with a difference of 35.7%. Moreover, the combination of RA and NEN further enhanced this separation, with the ‘High’ gene signature group achieving a 94.8% overall survival rate, compared to 51.8% in the ‘Low’ gene signature group, with a difference of 41.7%, suggesting that using uncoupler or the combination of RA + uncoupler could be beneficial to patients (Fig. 5G).

### Single-Cell RNA Sequencing Reveals Differentiation Dynamics in Neuroblastoma under Treatment

**Figure 6:**
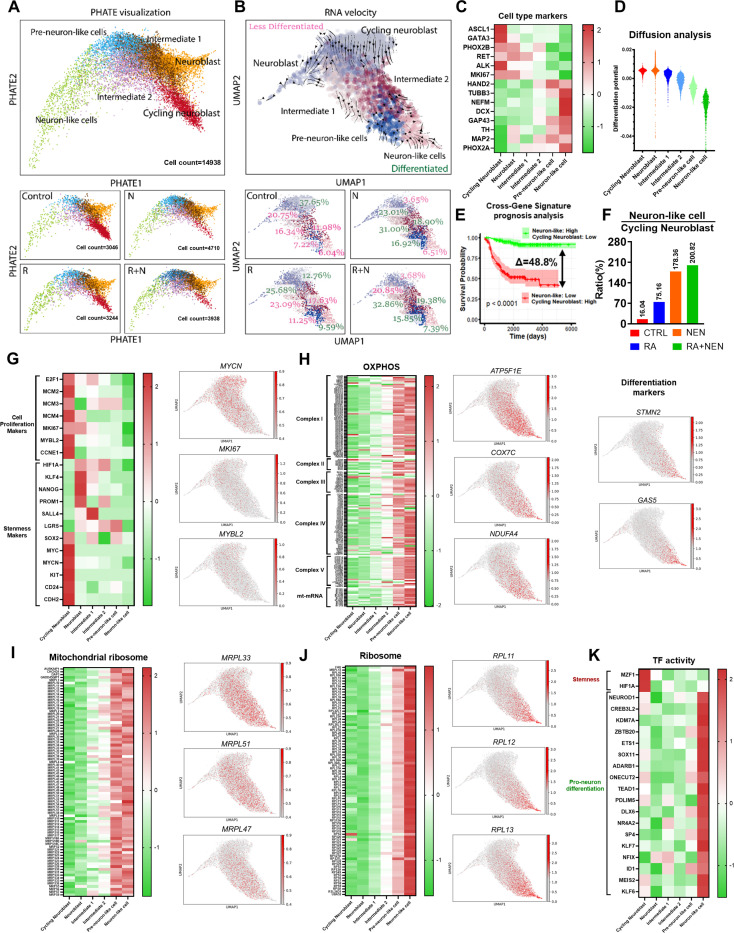
Single-cell RNA sequencing reveals differentiation dynamics induced by RA and uncoupler treatments. (**A**) PHATE visualization showing the differentiation trajectory from Cycling Neuroblasts to Neuron-like cells, illustrating distinct cell clusters at different stages of differentiation (Cycling Neuroblast, Neuroblast, Intermediate 1, Intermediate 2, Pre-neuron-like, and Neuron-like cells). (**B**) RNA velocity analysis highlights the directional differentiation process, with cells transitioning from less differentiated (Cycling Neuroblast) to more differentiated (Neuron-like cells). (**C**) Heatmap of differentiation stage markers indicating the expression patterns of key genes across different cell clusters. Cycling neuroblast markers include *ASCL1*, *GATA3*, *PHOX2B*, *RET*, *ALK*, and *MKI67*, while neuron markers include *HAND2*, *TUBB3*, *NEFM*, *DCX*, *GAP43*, *TH*, *MAP2*, and *PHOX2A*.(**D**) Diffusion analysis shows the transition probabilities across the cell populations, emphasizing the differentiation from Cycling Neuroblasts to Neuron-like cells. (**E**) Cross-gene signature prognosis analysis shows a significant improvement in survival probability associated with higher Neuron-like/Cycling Neuroblast ratios, highlighting the therapeutic relevance of promoting differentiation. (**F**) Bar graph comparing the ratio of Neuron-like cells to Cycling Neuroblasts across treatment conditions. (**G**) Heatmap of cell proliferation markers and stemness markers across cell clusters and treatments. (**H-K**) Heatmaps depicting the expression patterns of genes related to OXPHOS, mitochondrial ribosome, ribosome, and transcription factor activity, respectively, along the differentiation trajectory.

To further investigate the differentiation dynamics in neuroblastoma under RA and NEN treatment, we performed single-cell RNA sequencing using *in vivo* orthotopic neuroblastoma samples. To minimize batch effects and other biological interference, the experiment was conducted under the same conditions as previously described, with seven mice per group (Fig. 5A). Two mice from each group, selected based on similar treatment durations and tumor sizes ranging from 500 to 700 mm, were collected and dissociated for single-cell analysis (Figure S6A). A total of approximately 14,938 cells were analyzed and classified into six distinct clusters. Using previously reported markers for cycling neuroblasts—such as *ASCL1*, *GATA3*, *PHOX2B*, *RET*, *ALK*, and *MKI67* ^50,51^—we identified cycling neuroblasts and neuroblasts. Additionally, using neuron markers including *HADN2*, *TUBB3*, *NEFM*, *DCX*, *GAP43*, *TH*, *MAP2*, and *PHOX2A* ^52*–*59^, we identified pre-neuron-like cells and neuron-like cells. The remaining two clusters were assigned as intermediate 1 and 2 (Fig. 6C). In general, neuron-like cells are expected to be more differentiated than cycling neuroblasts. To validate this in our data, we performed RNA velocity analysis to determine the differentiation trajectory among the clusters. As expected, the trajectory arrows originated from cycling neuroblast and ended in neuron-like cells, confirming the differentiation hierarchy (Fig. 6B). However, we were unable to distinguish the differentiation status between intermediate 1 and intermediate 2. To address this, we performed diffusion map analysis across all clusters to assess their differentiation potential (Fig. 6D). The results suggest that intermediate 2 is more differentiated than intermediate 1 and further validate the overall differentiation hierarchy across all clusters (Fig. 6D, S6B).

To assess the potential correlation of cycling neuroblasts and neuron-like cells on prognosis, we used their marker genes to stratify patients based on the median Z-score for each group, respectively. Consistent with the observation that higher differentiation is associated with better prognosis in neuroblastoma, elevated cycling neuroblast markers were linked to worse prognosis, while elevated neuron-like cell markers were associated with improved prognosis (Figure S6C, 6D). Additionally, based on the median Z-score, patients were stratified into four groups: (1) High/Low, (2) Low/Low, (3) High/High, and (4) Low/High for neuron-like cells and cycling neuroblasts, respectively (Figure S6D). The highest prognosis was observed in the High/Low group with a survival rate of 91.9%, while the lowest prognosis was in the Low/High group with a survival rate of 42.1%, suggesting that a higher ratio of neuron-like cells to cycling neuroblasts is significantly associated with better prognosis (Fig. 6E, S6D). Single-cell RNA sequencing revealed a distinct progression of cellular states from low to high differentiation across treatment conditions. In the control group, 37.65% of cells were cycling Neuroblasts, while 20.75% were neuroblasts, and only 6.04% reached the fully differentiated neuron-like cell state. RA treatment reduced the cycling neuroblast population to 12.76% while increasing the proportions of more differentiated cells, including 25.68% neuroblasts, 23.09% intermediate 1 cells, and 9.59% neuron-like cells. NEN treatment further suppressed cycling neuroblasts to 3.65%, with increases in intermediate 1 to 31.00% and pre-neuron-like cells to 16.92%, though neuron-like cells remained low at 6.51%. Notably, the combination of RA and NEN resulted in the greatest reduction of cycling neuroblasts to 3.68% and further reduced neuroblasts (20.85%) compared to NEN alone (23.01%), with Intermediate 1 cells increasing to 32.86% and neuron-like cells to 7.39%, indicating a significant shift towards more differentiated cell types (Fig. 6B, S6E). Importantly, RA increased the ratio of neuron-like cells to cycling neuroblasts to 75.16%, NEN to 178.36%, and the combination of RA and NEN achieved the highest ratio of 200.82%, indicating better prognosis with the combined treatment (Fig. 6E, F).

Next, we investigated transcriptional hallmark dynamics along the differentiation trajectory, revealing distinct patterns in cell proliferation, stemness, and mitochondrial functions among the differentiation stages. Proliferation markers like *E2F1*, *MCM2*, *MKI67* and stemness markers such as *MYCN* and *CD24* were highly expressed in less differentiated states and decreased as cells progressed towards more mature, differentiated states, (Fig. 6G). Interestingly, a group of stemness markers, including *PROM1*, *NANOG*, and *KLF4*, showed low expression in cycling neuroblasts but were upregulated in neuroblasts, aligning with the presence of a cell population in the neuroblast stage with high differentiation potential (Fig. 6D, G). These markers later decreased as cells progressed towards more mature states. OXPHOS genes showed the opposite pattern, with their expression increasing significantly as cells differentiated, reflecting enhanced mitochondrial function in more mature cells (Fig. 6H). Mitochondrial ribosomal and translation factor genes, which support mitochondrial biogenesis, also increased along the differentiation trajectory (Fig. 6I), as did cytoplasmic ribosomal genes (Fig. 6J), highlighting the growing need for protein synthesis in transition from self-renewal to differentiation ^60^. Finally, transcription factor activity shifted from proliferation-related factors like *MZF1* and *HIF1A* in less differentiated states to differentiation-related factors like *NEUROD1* and *ASCL1* in more differentiated states (Fig. 6K). This progression demonstrates the transcriptional reprogramming that drives the shift from proliferation to differentiation.

## Discussion

The symbiogenesis theory, proposed in 1905, suggests that eukaryotic cells evolved by engulfing a free-living aerobic bacterium, which enabled efficient ATP production through oxidative phosphorylation, driving the evolution of multicellularity and cell differentiation^25^. This theory aligns with observations in developmental processes, such as neurodevelopment, where differentiation is associated with increased mitochondrial quantity and enhanced oxidative phosphorylation ^61^. Conversely, in the 1920s, Otto Warburg proposed that impaired mitochondrial respiration in differentiated cells causes them to rely on glycolysis instead of oxidative phosphorylation, leading to dedifferentiation and driving tumorigenesis ^62,63^. Thus, mitochondrial homeostasis directly regulates cell fate determination and is essential for maintaining cellular differentiation. Since many cancers exhibit reduced mitochondrial quality and quantity, restoring mitochondrial quality and quantity could be a promising strategy for cancer differentiation therapy.

RA has shown strong potential as a differentiation agent, achieving 90% remission and over 60% cure rates in Acute Promyelocytic Leukemia (APL). *In vitro* studies show that RA exerts significant effects on proliferation arrest, apoptosis, and differentiation in solid tumor cell lines. However, the outcomes from clinical trials have been less promising ^48,64^. Neuroblastoma (NB) is one of the most common solid cancer types in childhood, with over 600 cases per year in the U.S. In high-risk patients, disease relapse occurs frequently after surgery or chemotherapy and eventually becomes fatal. The effect of RA has been mostly studied in NB among solid tumors because it induces clear neuronal differentiation morphology and proliferation arrest in many NB cell lines. One clinical trial showed that RA significantly increased event-free survival in patients who took myeloablative therapy followed by autologous bone marrow transplantation (ABMT) or intensive chemotherapy ^35^. However, even in the ABMT + RA group, over 40% of the patients did not respond to treatment and died within five years ^65^. RA-responsive NB cells can develop RA resistance, particularly under hypoxic conditions^13,66^. Altogether, these studies underscore the need for a deeper understanding of cancer cell differentiation mechanisms and the development of more advanced differentiation strategy ^48,64^. Along with previous studies that focused on RA’s role in activating RA receptor-dependent transcription, we further expand its role in regulating mitochondrial biogenesis partly through upregulating nuclear-encoded mitochondria genes to induce differentiation. In our study, we demonstrated that RA strongly promotes mitochondrial biogenesis and elongation. Although RA treatment decreased the glucose flux into the pentose pathway for nucleotide synthesis, RA treatment alone failed to activate cellular respiration or TCA cycle flux. This is likely because dedifferentiated cancer cells reduce high ATP-consuming functions, leading to a lower overall ATP demand, consistent with empirical data showing that cancer cells need less ATP for growth and division compared to normal cells for maintenance ^67^. As a result, the excess mitochondrial quantity leads to ATP accumulation, which can inhibit ATP synthase activity. Since ATP syntheses use the proton gradient to generate ATP, reduced ATP synthesis leads to an elevated mitochondrial proton gradient, which in turn limits ETC activity. This restriction lowers the NAD^+^/NADH ratio, ultimately slowing or even partially reversing the TCA cycle through reductive carboxylation ^45^. TCA cycle metabolites are essential for maintaining the epigenetic landscape^38^ and differentiation state^15^. Therefore, a slow or dysregulated TCA cycle can block differentiation, leading to resistance to RA-based differentiation therapy.

To overcome this limitation, we employed mitochondrial uncouplers to create a pseudo-ATP demand, activating ETC and TCA cycle. Mitochondrial uncouplers are small molecules that act as cross-membrane proton transporters, dissipating the membrane potential and reactivating the ETC. This increases the NAD^+^/NADH ratio, reverses the Warburg effect, and improves mitochondrial quality. This increase in the NAD^+^/NADH ratio plays a pivotal role in accelerating key steps of the TCA cycle. When the NAD^+^/NADH ratio is reduced, the TCA cycle slows down, and the utilization of glucose and glutamine is diminished. However, glutamine is more resilient to lower NAD^+^/NADH levels, contributing to the “glutamine addiction” often seen in cancers ^68^. Isotope tracing using U-^13^C-glucose and U-^13^C-glutamine revealed that restoring mitochondrial quantity and quality with RA and uncoupler caused a metabolic transition, driven by the increased NAD^+^/NADH ratio, which rerouted glucose and glutamine from the pentose phosphate pathway towards oxidative phosphorylation. This transition was accompanied by a significant acceleration of the TCA cycle and reduced reliance on reductive carboxylation. Notably, the reprograming TCA cycle exhibited a higher preference for glucose as a primary substrate over glutamine, signifying a shift from biosynthesis to energy production. This data suggests that although differentiated cells may have a high ATP demand, elevated ATP levels do not promote differentiation. Differentiation requires a high ATP turnover rate to lower the mitochondrial membrane potential, enabling ETC activation and accelerating the TCA cycle, which in turn facilitates the epigenetic remodeling necessary for differentiation to occur. While differentiated cells typically possess more mitochondria than stem/progenitor cells ^15,40^—suggesting that the capacity to produce ample ATP was crucial for differentiation during evolution—maintaining this differentiated state in modern multicellular organisms requires not just ATP production but also continuous ATP consumption and sustained ETC activity.

Restoring mitochondrial function with both RA and uncouplers promotes neuroblastoma differentiation and triggers cell cycle arrest. While both neurite length and number increase during differentiation, the rise in neurite number plays a more significant role in determining the extent of differentiation. This suggests that neurite number may increase initially as the neuron forms multiple processes, and neurite length becomes more prominent as the neuron matures and elongates its processes for connectivity.

Previous studies have shown that mitochondrial number and activity increase during the normal differentiation process ^15,40^. To determine whether mitochondrial activity is merely associated with differentiation or is actually essential for tumor differentiation, we used both pharmacological and genetic tools to inhibit mitochondrial respiration. A complex I Inhibitor rotenone could attenuate this differentiation, suggesting that mitochondrial reparation is necessary for cell differentiation. Furthermore, we found that Rho-0 cells generated by either chemical or genetic intervention were unable to display differentiation upon RA + NEN combination treatment. Interestingly, the neuroblastoma Rho-0 cells form clusters, a sign of loss of contact inhibition, which is associated with enhanced malignancy and invasiveness ^69^. Our studies demonstrated that restoring mitochondrial quality and quantity is both necessary and sufficient to induce differentiation in cancer.

As a pediatric cancer, neuroblastoma serves as an ideal model for studying how disruptions in early development and differentiation can lead to tumorigenesis. Recently, single-cell RNA sequencing (scRNA-seq) has been used to identify the cell of origin in neuroblastoma and to reveal lineage alterations occurring during tumorigenesis^70–76^. These studies identified precursor/progenitor lineages from which neuroblastoma may originate; however, no existing studies have yet shown lineage transition changes in response to differentiation therapy. Using single-cell sequencing, we further validated the differentiation-inducing effects of RA and NEN. RA alone promoted cellular differentiation, while NEN effectively reduced the stemness population. Notably, the combination of RA and NEN demonstrated the strongest effect, inducing the most pronounced differentiation while simultaneously eliminating stemness characteristics, in line with the histological analysis. Interestingly, stemness markers such as *PROM1*, *NANOG*, and *KLF4* exhibited low expression in cycling neuroblasts but were upregulated in neuroblasts, consistent with the presence of a highly differentiation-capable population at this stage. This could be explained by the fact that certain cancers may exist in a transdifferentiated state ^77^, requiring a reversion to a stem-like state before proper differentiation can proceed. This suggests that RA and NEN treatments may guide cells back through this critical state before driving full differentiation, Importantly, the upregulation of OXPHOS genes, mitochondrial ribosomal proteins, and translation factors along the differentiation trajectory supports our conclusion that mitochondrial homeostasis is crucial for successful differentiation.

The Warburg effect was discovered over 100 years ago. In 1956, Otto Warburg proposed a two-step process during tumorigenesis: (a) an irreversible impairment of mitochondrial respiration and (b) a metabolic shift toward glycolysis that drives the dedifferentiation of normal cells into “wildly growing cancer cells.” In recent decades, substantial efforts have been directed toward targeting hyperactive glycolysis or inhibiting the relatively slow mitochondrial respiration in cancer cells. However, these strategies have largely failed due to limited efficacy and considerable toxicity to healthy tissues ^2^. In our study, we demonstrated that mitochondrial impairment is, in fact, reversible. Mitochondrial dysfunction arises because dedifferentiated cancer cells had low ATP consumption, thereby limiting mitochondrial function. By employing uncouplers to create a pseudo-ATP demand, we were able to reactivate mitochondrial function and promote the differentiation of tumor cells. More importantly, our data revealed that the Warburg effect extends beyond cell proliferation and plays a crucial role in determining cell fate. Additionally, future studies could explore whether these differentiated neurons within tumors can develop intrinsic energy-consuming activities once treatment is withdrawn.

### Study limitations:

The combination of RA and the uncoupler achieved only partial differentiation in vivo, likely due to heterogeneity within the tumor microenvironment. Combining spatial transcriptomic and metabolic analyses could help uncover these underlying factors. In addition, there are notable limitations in using current mitochondrial uncouplers due to their poor bioavailability and the narrow therapeutic window within which they function effectively. Future research should focus on identifying novel uncouplers with improved bioavailability and broader therapeutic windows, allowing for safer and more effective clinical applications. Further investigation is also needed to refine dosing and delivery methods for improved therapeutic efficacy. Nevertheless, our proof-of-principle study highlights the crucial role of mitochondrial homeostasis in cancer differentiation and offers a promising avenue for developing therapies that promote tumor differentiation through mitochondrial reprogramming.

## Materials and Methods

### Cell Culture and Reagents

MYCN-amplified neuroblastoma cell lines SK-N-BE(2), SY-SH5Y, CHP134 and LAN-5 were obtained from Dr. John M. Maris’s laboratory (Children’s Hospital of Philadelphia) in 2016. A certificate of analysis is available from Dr. Maris’s group. All cell lines were tested every two months and found negative for Mycoplasma using the MycoAlert Mycoplasma Detection Kit (Lonza). The last testing was performed in September 2024. Cell lines used for experiments were within 5 to 15 passages. The authors did not authenticate these cell lines. All cell lines were cultured in DMEM/F12 medium (Gibco Labs; 11320033) supplemented with 1% penicillin–streptomycin (Gibco; 15140122), 10% FBS (Sigma-Aldrich; F0926), and 1 mmol/L glutamine (Gibco; 25030081). NEN (#17118), BAM15 (#17811) 13-cis-retinoic acid (#21648) and was obtained from Cayman. The Oligomycin, FCCP and Rotenone/Antimycin A mixture were obtained from were obtained from the Seahorse XF Real-Time ATP Rate Assay Kit (103592–100, Agilent) and the Seahorse XF Cell Mito Stress Test Kit (#103015–100, Agilent). Anti-β-tubulin III antibody (#5568S) and anti-Actin antibody (#3700S) were purchased from Cell Signaling Technology. Anti-TFAM antibody (#MA5–16148) was purchased from Invitrogen. The horseradish peroxidase-conjugated secondary antibody and Alexa Fluor 594-conjugated goat anti-rabbit IgG (H+L) cross-adsorbed secondary antibody (#A-11012) were obtained from Invitrogen.

### Protein Extraction and Immunoblotting

Cells were washed with ice-cold PBS buffer (Invitrogen; 20012050) and lysed with RIPA Lysis and Extraction Buffer (Thermo Scientific; 89900) supplemented with 1% Halt Protease inhibitor Cocktail (Thermo Fisher Scientific; 78443) for 10 minutes. The lysate was centrifuged at 12,000 rpm for 20 minutes at 4°C, and the supernatant containing proteins was collected. Protein concentration was determined using the BCA assay (Thermo Fisher Scientific, 23227). Immunoblotting was performed as previously described, with blots representing at least two independent experiments.

### RNA Isolation, Reverse Transcription, and Real-Time Quantitative PCR

Total RNA was isolated from 60 mm tissue culture plates using the TRIzol Reagent (Invitrogen; 15596026) following the manufacturer’s protocol. Three micrograms of total RNA were used in a reverse transcription reaction with the iScript cDNA Synthesis Kit (Bio-Rad). RT-qPCR amplification was performed using the Prism 7900 Sequence Detection System (Applied Biosystems) and TaqMan Gene Expression Assays (Applied Biosystems). Gene expression levels were normalized to 18S ribosomal RNA. Data are presented as mean ± SD across three RT-qPCR reactions, and representative values from three independent experiments are shown.

### Mitochondrial imaging

The pMXs-3XHA-EGFP-OMP25 (#83356) plasmid was obtained from Addgene and introduced into SK-N-BE(2) and CHP134 cells. For the quantification of mitochondrial GFP intensity, cells were seeded in 96-well plates (Corning; #3603) using FluoroBrite^™^ DMEM (Gibco; #A1896701) to reduce autofluorescence. After 24 hours of incubation, 1 μM RA, 1 μM NEN, and their combination were added to the wells, and the plate was transferred to the Agilent Cytation 5 cell imaging multimode reader, maintained at 37°C with 5% CO. Bright-field and GFP channel images were acquired every hour for up to 72 hours. At the endpoint, Hoechst stain (Thermo; #33342) was added to the plate to quantify cell numbers for GFP signal normalization. For mitochondrial morphology imaging, cells were seeded in Nunc^™^ Lab-Tek^™^ II Chambered Coverglass (Thermo Scientific^™^; #155409) using FluoroBrite^™^ DMEM (Gibco; #A1896701) to minimize autofluorescence. After 24 hours of incubation, 1 μM RA, 1 μM NEN, and their combination were added to the wells, and mitochondrial morphology was captured using a Zeiss LSM 880 confocal microscope with a 63x oil immersion lens.

### Seahorse assay

Two sets of experiments were performed. In the first experiment, SK-N-BE(2) and CHP134 cells were treated with 1 μM RA, 1 μM NEN, or their combination for 72 hours. After this pretreatment, approximately 15,000 live cells were replated into each well of a Seahorse cell culture microplate, incubated overnight, and then subjected to a standard mitochondrial stress test to assess mitochondrial function following the manufacturer’s instructions. In the second experiment, 15,000 live cells were seeded directly into a Seahorse cell culture plate to investigate the effects of various uncouplers. FCCP, NEN, and BAM15 (at 10x the indicated concentrations) were added to port A of the Seahorse cartridges. The compounds were injected after baseline measurements, and the assay continued for 96 minutes. At the end of both Seahorse assays, Hoechst stain (Thermo; #33342) was injected through port D to quantify cell numbers, using the Biotek Cytation 5 Cell Imaging Multimode Reader for analysis. The cell count data were subsequently used to normalize the Seahorse assay results.

### PCR Based Determination of Mitochondrial DNA Copy Number

SK-N-BE(2) and CHP134 cells were treated with 1 μM RA for 72 hours, after which DNA was extracted using the DNeasy Blood and Tissue Kit (Qiagen; #69506) following the manufacturer’s instructions. The mtDNA copy number was quantified based on a previously established method. In brief, 50 ng of DNA served as the template, and 0.4 μM primers were used with PowerUp^™^ SYBR^™^ Green Master Mix (Applied Biosystems^™^, # A25918) for qPCR. To calculate the mtDNA copy number, the difference in Ct values was first determined for the ND1/actin pair (ΔCt1 = Ct for actin - Ct for ND1) and the TL1/actin pair (ΔCt2 = Ct for actin - Ct for TL1). Similarly, B2M was used as another genomic reference gene, with ΔCt3 and ΔCt4 calculated for the ND1/B2M and TL1/B2M pairs, respectively. The 2^ΔCt values were then computed to represent the mtDNA copy number.

### Next-Generation RNA Sequencing and Bioinformatics Analysis

Total RNA from treatment groups (control and NEN-treated; n = 3) was extracted using TRIzol Reagent (Invitrogen; 15596026) following the manufacturer’s instructions. Libraries were constructed and subjected to 150 bp paired-end sequencing on an Illumina platform at Novogene. RNA-seq analysis was conducted using the kallisto and sleuth analytical pipelines. A transcript index was generated based on Ensembl version 67 of the hg19 human reference genome. Paired-end RNA-seq reads were pseudo-aligned using kallisto (v0.42.4) with 100 bootstraps (-b 100) to estimate the variance of transcript abundances. Transcript-level estimates were aggregated to transcripts per million (TPM) for each gene, with Ensembl gene names assigned using biomaRt. Differential gene expression analysis was performed using the sleuth R package across pairwise groups, with significant hits identified based on a sleuth q-value < 0.05 and absolute log2 fold change > 0.693. Gene set enrichment analysis (GSEA) was employed to identify significantly enriched pathways by comparing the normalized RNA-seq TPM dataset between groups against the molecular signatures database (MSigDB v7.4).

To further assess the combined effects of RA and NEN, a focused analysis was performed on a set of 21 genes specifically induced by the RA+NEN treatment. The average z-scores for these 21 genes were calculated, and patients were stratified based on these scores to evaluate transcriptional changes and their correlation with clinical outcomes. Subsequently, a broader bioinformatic analysis was conducted to assess the impact of RA, NEN, or their combination on patient outcomes. Patients were stratified into four groups based on the average z-scores for upregulated and downregulated gene lists: (1) high upregulated/low downregulated, (2) low upregulated/low downregulated, (3) high upregulated/high downregulated, and (4) low upregulated/high downregulated. These groups were then used to generate survival curves, linking gene expression profiles with patient survival outcomes. Kaplan–Meier analysis was conducted on neuroblastoma patient datasets from R2, focusing on the *MYCN*, *NTRK1*, *S100B*, and *NF1* genes.

### Neurite Outgrowth Assay

Neurite outgrowth quantification was conducted as previously described ^13,23^. Briefly, 1 × 10^4^ SK-N-BE(2), SH-SY5Y, CHP134, and LAN-5 neuroblastoma cells per well were seeded into a 12-well plate. After overnight incubation, cells were treated with 1 μM RA, 1 μM NEN, or their combination, with or without 0.05 μM rotenone, for 72 hours. Images were captured using a Leica Dmi8 Fluorescent Microscope in phase contrast mode at 10× magnification. Neurite lengths were traced and quantified using the NeuronJ plugin for ImageJ. For each sample, the total neurite length was measured and normalized to the number of cell bodies. Data are presented as mean ± SD across three biological replicates.

### Immunofluorescence Staining

SK-N-BE(2) and NB16 cells were seeded into 8-chamber slides (Thermo Fisher Scientific; 154534) at 6 × 10^3 cells/well overnight and subjected to the indicated treatment for 72 hours. Cells were fixed with 4% paraformaldehyde in PBS with 0.1% Tween-20 at room temperature for 30 minutes, followed by permeabilization with 0.1% Triton X-100 in PBS at room temperature for 10 minutes. Cells were washed with PBS twice and blocked with 2.5% horse serum in PBS at room temperature for 1 hour. Immunofluorescence staining was performed with primary antibody β3-Tubulin (CST; 5568) at 4°C overnight. After two washes with PBS, cells were incubated with Alexa Fluor 594-conjugated anti-rabbit secondary antibody (Life Technologies) at room temperature for 1 hour, followed by staining with DAPI (Vector Laboratories, H-1800–2). Images were acquired using a Leica Dmi8 microscope. Data are representative of two independent experiments.

### Cell proliferation assay

For synergy testing, the Chou-Talalay method was utilized to evaluate the synergistic, additive, or antagonistic effects of drug combinations. SK-N-BE(2) and CHP134 cells were treated with varying concentrations of RA, NEN, and their combinations. Cell proliferation was assessed after 72 hours, with Combination index (CI) values calculated using CompuSyn software, where CI < 1 indicates synergism, CI = 1 indicates an additive effect, and CI > 1 indicates antagonism. For the replating assay, cells were treated with RA, NEN, and their combinations for 4 days, after which an equal number of viable cells were replated into 96-well plates without further treatment. Cell numbers were then assessed at 48 and 96 hours post-replating, using the Cytation 5 imaging system with Hoechst stain (Thermo; #33342), following the manufacturer’s protocols. All experiments were conducted in six replicates.

### Metabolic Analysis

Metabolomics and isotope tracing analyses were performed using an Agilent 1290 Infinity Liquid Chromatography (LC) System coupled to a Q-TOF 6545 mass spectrometer (MS; Agilent).Chromatography included standardized HILIC methodology previously described using the InfinityLab Poroshell 120 HILIC-Z column (Agilent; 683775–924) and spectral library with retention time information ^78^. Targeted analysis, isotopologue extraction, and natural isotope abundance correction were performed using Agilent Profinder B.10.00, as previously described. Data are presented as mean ± SD across three biological replicates.

### Stable Isotope Tracing Analysis

SK-N-BE(2) and CHP134 cells were pretreated with DMSO, 1 μM RA, 1 μM NEN, or their combination for 72 hours. Following pretreatment, glucose isotope tracing was performed by replacing the culture medium with medium (USBiological; D9807–02) supplemented with 1.2 g/L sodium bicarbonate and 17.5 mmol/L U-13C-glucose (Cambridge Isotope Laboratories; CLM-1396–10) for 5 hours. For glutamine isotope tracing, the medium was replaced with DMEM/F-12 without glutamine (Gibco; 21331020) and supplemented with 4 mmol/L U-13C-glutamine (Cambridge Isotope Laboratories; CLM-1822-H) and 10% dialyzed FBS (Gibco; 26400044) for 5 hours. Data are presented as mean ± SD across three biological replicates.

### Generation of ρ0 cells for neuroblastoma cell

SK-N-BE(2) and CHP134 neuroblastoma cells were cultured in DMEM/F12 medium supplemented with 1% sodium pyruvate (100 mM), 50 μg/ml uridine, and 50 ng/ml ethidium bromide (EtBr) to deplete mitochondrial DNA and generate ρ0 cells. The depletion process took approximately one month, during which the cells were continuously maintained under these conditions until ρ0 cells were successfully established. Additionally, gRNA targeting TFAM (Gene ID: 7019) was cloned into the lentiCRISPR v2 vector (Addgene; #52961), packaged, and used to infect CHP134 cells. Infected cells were selected with 1.5 ng/ml puromycin for 2 weeks and then maintained in 0.75 ng/ml puromycin. The generation of ρ0 cells was confirmed through Seahorse metabolic analysis and mtDNA copy number qualification.

### Mouse Orthotopic Neuroblastoma Model

All mouse procedures were conducted in accordance with Stanford University guidelines for the care and use of animals and were approved by the Institutional Animal Care and Use Committee. The experiments were performed using 7-week-old male nude mice (Taconic). General anesthesia was administered using isoflurane inhalation for all procedures, including ultrasound measurements. Orthotopic tumors were established within the adrenal gland as previously described. A transverse incision was made on the left flank to expose the left adrenal gland, and 1 mL of PBS containing 10^5 SK-N-BE(2) cells was injected into the gland using a 30G needle. The fascia and skin were closed in separate layers. Tumor formation was monitored by non-invasive ultrasound, and diet intervention began once the tumor volume reached 5 to 20 mm^3. The mice were provided with one of the following diets obtained from Research Diets, Inc.: a control diet (#D11112201), a diet containing 83.37 ppm RA (#D22040408), a diet with 2,000 ppm NEN (#D20101601)^45,79,80^, or a combination diet of RA and NEN (#D22040411). The animals were euthanized when the tumor volume exceeded 1,000 mm^3.

### Monitoring Tumor Growth with High-Frequency Ultrasound

Mice were secured in a prone position, and a Vevo 2100 sonographic probe (Visual Sonics) was applied to the left flank to locate the left adrenal gland and the tumor. Tumor monitoring by ultrasound was performed twice weekly. Serial cross-sectional images (0.076 mm between images) were taken, and tumor volume was measured using a 3D reconstruction tool (VevoLab 5.8.2).

### Histologic and Immunohistochemical Examination of Mouse Orthotopic Tumors

Hematoxylin and eosin (H&E) and immunohistochemically stained sections were prepared from formalin-fixed and paraffin-embedded blocks of mouse orthotopic tumors. Immunostaining involved heating unstained sections for 30 minutes in Bond Epitope Retrieval Solution 2 (No. AR9640; Leica Biosystems Newcastle Ltd.) and incubating them with anti-human N-Myc rabbit monoclonal antibody (1:300 dilution; Cell Signaling Technology, #51705), anti-human S100β rabbit monoclonal antibody (Cell Marque, #EP32), anti-human TrkA rabbit monoclonal antibody (Abcam, #EPR17341) followed by counter-staining with hematoxylin.

### Single cell analysis of the neuroblastoma xenograft

Tumors with volumes ranging from 500 to 800 mm, were collected from each group (CTRL, RA, NEN, and RA+NEN diet) within approximately 2 weeks of treatment. Two tumors per group were harvested for single-cell suspension preparation. Tumors were dissociated using the Tumor Dissociation Kit (Miltenyi; #130–095-929) in combination with the gentleMACS^™^ Octo Dissociator (Miltenyi; #130–096-427). The resulting single-cell suspensions were prepared for library construction using the PIPseq^™^ V T10 3’ Single Cell RNA Kit. Sequencing was performed by Novogene, and the raw single-cell RNA sequencing (scRNA-seq) FASTQ data were processed using Pipseeker(Fluent Bioscience) and Cell Ranger (10x Genomics) to generate gene expression count matrices and unspliced read counts necessary for RNA velocity analyses. Subsequent data processing was performed with the Python package Scanpy. Initial filtering excluded cells expressing fewer than 200 genes and genes detected in fewer than 3 cells. Quality control steps removed cells with n_genes_by_counts more than 400 and removed cells exhibiting mitochondrial gene expression exceeding 5% of total counts. Clustering was conducted using the Louvain algorithm within Scanpy, and cell types were annotated based on marker genes identified via the ‘rank_genes_groups’ function. Mouse cell clusters were then identified and excluded to retain only human cells for downstream analyses. RNA velocity analyses were performed using Velocyto and scVelo packages. Pathway enrichment analysis was conducted with QIAGEN’s Ingenuity Pathway Analysis software. Diffusion map analyses were implemented using the ‘diffmap’ function in Scanpy. Transcription factor activity inference was carried out using the Python package pySCENIC.

### Statistical Analysis

Cell proliferation and LC/MS experiments were analyzed using three biological replicates. Results are presented as mean ± SD. Data from mouse orthotopic neuroblastoma experiments are presented as mean ± SEM. Statistical significance between groups was assessed using Student’s t-tests and one- or two-way ANOVA tests (two-tailed; unequal variance).

## Supplementary Material

Supplement 1

## References

[1] Vander Heiden M. G., Cantley L. C. & Thompson C. B. Understanding the Warburg effect: the metabolic requirements of cell proliferation. science 324, 1029–1033 (2009). 19460998 10.1126/science.1160809 PMC2849637

[2] Thompson C. B. . A century of the Warburg effect. Nat Metab 5, 1840–1843, doi:10.1038/s42255-023-00927-3 (2023). 37990075

[3] Warburg O. On the origin of cancer cells. Science 123, 309–314 (1956). 13298683 10.1126/science.123.3191.309

[4] DeBerardinis R. J. & Chandel N. S. We need to talk about the Warburg effect. Nature Metabolism 2, 127–129, doi:10.1038/s42255-020-0172-2 (2020). 32694689

[5] Reznik E. Mitochondrial DNA copy number variation across human cancers. Elife 5, doi:10.7554/eLife.10769 (2016). PMC4775221 26901439

[6] Reznik E., Wang Q., La K., Schultz N. & Sander C. Mitochondrial respiratory gene expression is suppressed in many cancers. Elife 6, doi:10.7554/eLife.21592 (2017). PMC5243113 28099114

[7] Bartman C. R. Slow TCA flux and ATP production in primary solid tumours but not metastases. Nature, doi:10.1038/s41586-022-05661-6 (2023). PMC10288502 36725930

[8] Shen Y. Mitochondrial ATP generation is more proteome efficient than glycolysis. Nat Chem Biol, doi:10.1038/s41589-024-01571-y (2024). PMC11925356 38448734

[9] Chen H. & Chan D. C. Mitochondrial Dynamics in Regulating the Unique Phenotypes of Cancer and Stem Cells. Cell Metab 26, 39–48, doi:10.1016/j.cmet.2017.05.016 (2017). 28648983 PMC5539982

[10] Garcia-Bermudez J. . Aspartate is a limiting metabolite for cancer cell proliferation under hypoxia and in tumours. Nature Cell Biology 20, 775-+, doi:10.1038/s41556-018-0118-z (2018). 29941933 PMC6030478

[11] Sullivan L. B. . Supporting Aspartate Biosynthesis Is an Essential Function of Respiration in Proliferating Cells. Cell 162, 552–563, doi:10.1016/j.cell.2015.07.017 (2015). 26232225 PMC4522278

[12] Birsoy K. . An Essential Role of the Mitochondrial Electron Transport Chain in Cell Proliferation Is to Enable Aspartate Synthesis. Cell 162, 540–551, doi:10.1016/j.cell.2015.07.016 (2015). 26232224 PMC4522279

[13] Li Y. . Acetate supplementation restores chromatin accessibility and promotes tumor cell differentiation under hypoxia. Cell Death Dis 11, 102, doi:10.1038/s41419-020-2303-9 (2020). 32029721 PMC7005271

[14] Jiang H. W., Jedoui M. & Ye J. B. The Warburg effect drives dedifferentiation through epigenetic reprogramming. Cancer Biology & Medicine 20, 891–897, doi:10.20892/j.issn.2095-3941.2023.0467 (2023). PMC10845936 38318838

[15] Li A. M. & Ye J. B. Deciphering the Warburg Effect: Metabolic Reprogramming, Epigenetic Remodeling, and Cell Dedifferentiation. Annual Review of Cancer Biology 8, 35–58, doi:10.1146/annurev-cancerbio-062822-120857 (2024).

[16] Zhao Y. . In vivo monitoring of cellular energy metabolism using SoNar, a highly responsive sensor for NAD(+)/NADH redox state. Nat Protoc 11, 1345–1359, doi:10.1038/nprot.2016.074 (2016). 27362337

[17] Metallo C. M. . Reductive glutamine metabolism by IDH1 mediates lipogenesis under hypoxia. Nature 481, 380–384, doi:10.1038/nature10602 (2011). 22101433 PMC3710581

[18] Mullen A R. . Reductive carboxylation supports growth in tumour cells with defective mitochondria. Nature 481, 385–388, doi:10.1038/nature10642 (2011). 22101431 PMC3262117

[19] Wise D. R. . Hypoxia promotes isocitrate dehydrogenase-dependent carboxylation of alpha-ketoglutarate to citrate to support cell growth and viability. Proceedings of the National Academy of Sciences of the United States of America 108, 19611–19616, doi:10.1073/pnas.1117773108 (2011). 22106302 PMC3241793

[20] Jiang H. Mitochondrial uncoupling inhibits reductive carboxylation in cancer cells. Mol Cancer Res, doi:10.1158/1541-7786.MCR-23-0049 (2023). PMC10592403 37358566

[21] Intlekofer A M. . Hypoxia Induces Production of L-2-Hydroxyglutarate. Cell Metab 22, 304–311, doi:10.1016/j.cmet.2015.06.023 (2015). 26212717 PMC4527873

[22] Oldham W. M., Clish C. B., Yang Y. & Loscalzo J. Hypoxia-Mediated Increases in L-2-hydroxyglutarate Coordinate the Metabolic Response to Reductive Stress. Cell Metab 22, 291–303, doi:10.1016/j.cmet.2015.06.021 (2015). 26212716 PMC4526408

[23] Jiang H. . Mitochondrial uncoupling induces epigenome remodeling and promotes differentiation in neuroblastoma. Cancer Research 83, 181–194, doi:10.1158/0008-5472.Can-22-1029 (2023). 36318118 PMC9851961

[24] Shim E. H. . L-2-Hydroxyglutarate: an epigenetic modifier and putative oncometabolite in renal cancer. Cancer Discov 4, 1290–1298, doi:10.1158/2159-8290.CD-13-0696 (2014). 25182153 PMC4286872

[25] Li A. M. & Ye J. Deciphering the Warburg Effect: Metabolic Reprogramming, Epigenetic Remodeling, and Cell Dedifferentiation. Annual Review of Cancer Biology 8, 35–38, doi:10.1146/annurev-cancerbio-062822-120857 (2024).

[26] Kinnaird A., Zhao S., Wellen K. E. & Michelakis E. D. Metabolic control of epigenetics in cancer. Nat Rev Cancer 16, 694–707, doi:10.1038/nrc.2016.82 (2016). 27634449

[27] Maris J. M., Hogarty M. D., Bagatell R. & Cohn S. L Neuroblastoma. Lancet 369, 2106–2120, doi:10.1016/S0140-6736(07)60983-0 (2007). 17586306

[28] Matthay K. K. . Neuroblastoma. Nature Reviews Disease Primers 2, 16078, doi:10.1038/nrdp.2016.78 (2016). 27830764

[29] Shimada H. . The International Neuroblastoma Pathology Classification (the Shimada system). Cancer 86, 364–372 (1999). 10421273

[30] Sidell N. Retinoic acid-induced growth inhibition and morphologic differentiation of human neuroblastoma cells in vitro. J Natl Cancer Inst 68, 589–596 (1982). 7040765

[31] Amatruda T. T. 3rd, Sidell N., Ranyard J. & Koeffler H. P. Retinoic acid treatment of human neuroblastoma cells is associated with decreased N-myc expression. Biochem Biophys Res Commun 126, 1189–1195, doi:10.1016/0006-291x(85)90311-0 (1985). 3977910

[32] Thiele C. J., Reynolds C. P. & Israel M. A. Decreased expression of N-myc precedes retinoic acid-induced morphological differentiation of human neuroblastoma. Nature 313, 404–406, doi:10.1038/313404a0 (1985). 3855502

[33] Smith A. G., Popov N., Imreh M., Axelson H. & Henriksson M. Expression and DNA-binding activity of MYCN/Max and Mnt/Max during induced differentiation of human neuroblastoma cells. J Cell Biochem 92, 1282–1295, doi:10.1002/jcb.20121 (2004). 15258910

[34] Biedler J L., Helson L. & Spengler B A. Morphology and growth, tumorigenicity, and cytogenetics of human neuroblastoma cells in continuous culture. Cancer Res 33, 2643–2652 (1973). 4748425

[35] Matthay K. K. . Treatment of high-risk neuroblastoma with intensive chemotherapy, radiotherapy, autologous bone marrow transplantation, and 13-cis-retinoic acid. Children’s Cancer Group. N Engl J Med 341, 1165–1173, doi:10.1056/NEJM199910143411601 (1999). 10519894

[36] Tang X. H. & Gudas L. J. Retinoids, retinoic acid receptors, and cancer. Annu Rev Pathol 6, 345–364, doi:10.1146/annurev-pathol-011110-130303 (2011). 21073338

[37] Huang M. E. . Use of All-Trans Retinoic Acid in the Treatment of Acute Promyelocytic Leukemia. Blood 72, 567–572, doi:DOI 10.1182/blood.V72.2.567.567 (1988). 3165295

[38] Lu C. & Thompson C. B. Metabolic regulation of epigenetics. Cell Metab 16, 9–17, doi:10.1016/j.cmet.2012.06.001 (2012). 22768835 PMC3392647

[39] Kinnaird A., Zhao S., Wellen K. E. & Michelakis E. D. Metabolic control of epigenetics in cancer. Nature Reviews Cancer 16, 694–707, doi:10.1038/nrc.2016.82 (2016). 27634449

[40] Shyh-Chang N., Daley G Q. & Cantley L C. Stem cell metabolism in tissue development and aging. Development 140, 2535–2547, doi:10.1242/dev.091777 (2013). 23715547 PMC3666381

[41] Chen W. W., Freinkman E., Wang T., Birsoy K. & Sabatini D. M. Absolute Quantification of Matrix Metabolites Reveals the Dynamics of Mitochondrial Metabolism. Cell 166, 1324–1337 e1311, doi:10.1016/j.cell.2016.07.040 (2016). 27565352 PMC5030821

[42] Kenwood B. M. . Identification of a novel mitochondrial uncoupler that does not depolarize the plasma membrane. Mol Metab 3, 114–123, doi:10.1016/j.molmet.2013.11.005 (2014). 24634817 PMC3953706

[43] Chou T.-C. Drug Combination Studies and Their Synergy Quantification Using the Chou-Talalay Method. Cancer Research 70, 440–446, doi:10.1158/0008-5472.Can-09-947 (2010). 20068163

[44] King M. P. & Attardi G. Human cells lacking mtDNA: repopulation with exogenous mitochondria by complementation. Science 246, 500–503 (1989). 2814477 10.1126/science.2814477

[45] Jiang H. . Mitochondrial Uncoupling Induces Epigenome Remodeling and Promotes Differentiation in Neuroblastoma. Cancer Res 83, 181–194, doi:10.1158/0008-5472.CAN-22-1029 (2023). 36318118 PMC9851961

[46] Dzieran J. . MYCN-amplified neuroblastoma maintains an aggressive and undifferentiated phenotype by deregulation of estrogen and NGF signaling. Proc Natl Acad Sci U S A 115, E1229–E1238, doi:10.1073/pnas.1710901115 (2018). 29374092 PMC5819392

[47] Finklestein J. Z. . 13-cis-retinoic acid (NSC 122758) in the treatment of children with metastatic neuroblastoma unresponsive to conventional chemotherapy: report from the Childrens Cancer Study Group. Med Pediatr Oncol 20, 307–311, doi:10.1002/mpo.2950200407 (1992). 1608352

[48] Smith M. A. . Phase I and pharmacokinetic evaluation of all-trans-retinoic acid in pediatric patients with cancer. J Clin Oncol 10, 1666–1673, doi:10.1200/JCO.1992.10.11.1666 (1992). 1403049

[49] Villablanca J. G.. . Phase I trial of 13-cis-retinoic acid in children with neuroblastoma following bone marrow transplantation. J Clin Oncol 13, 894–901, doi:10.1200/JCO.1995.13.4.894 (1995). 7707116

[50] Ponzoni M. . Recent advances in the developmental origin of neuroblastoma: an overview. J Exp Clin Cancer Res 41, 92, doi:10.1186/s13046-022-02281-w (2022). 35277192 PMC8915499

[51] Jansky S. . Single-cell transcriptomic analyses provide insights into the developmental origins of neuroblastoma. Nat Genet 53, 683–693, doi:10.1038/s41588-021-00806-1 (2021). 33767450

[52] Lo L., Morin X., Brunet J.-F. & Anderson D. J. Specification of neurotransmitter identity by Phox2 proteins in neural crest stem cells. Neuron 22, 693–705 (1999). 10230790 10.1016/s0896-6273(00)80729-1

[53] Gleeson J. G., Lin P T., Flanagan L A. & Walsh C A. Doublecortin is a microtubule-associated protein and is expressed widely by migrating neurons. Neuron 23, 257–271 (1999). 10399933 10.1016/s0896-6273(00)80778-3

[54] Katsetos C. D., Legido A., Perentes E. & Mörk S. J. Class III β-tubulin isotype: a key cytoskeletal protein at the crossroads of developmental neurobiology and tumor neuropathology. Journal of child neurology 18, 851–866 (2003). 14736079 10.1177/088307380301801205

[55] Daadi M. M. & Weiss S. Generation of tyrosine hydroxylase-producing neurons from precursors of the embryonic and adult forebrain. J Neurosci 19, 4484–4497, doi:10.1523/jneurosci.19-11-04484.1999 (1999). 10341249 PMC6782621

[56] Maniero C.. . NEFM (Neurofilament Medium) Polypeptide, a Marker for Zona Glomerulosa Cells in Human Adrenal, Inhibits D1R (Dopamine D1 Receptor)-Mediated Secretion of Aldosterone. Hypertension 70, 357–364, doi:10.1161/HYPERTENSIONAHA.117.09231 (2017). 28584012

[57] Schmidt M.. . The bHLH transcription factor Hand2 is essential for the maintenance of noradrenergic properties in differentiated sympathetic neurons. Developmental Biology 329, 191–200, doi:10.1016/j.ydbio.2009.02.020 (2009). 19254708 PMC2746555

[58] Soltani M. H. . Microtubule-Associated Protein 2, a Marker of Neuronal Differentiation, Induces Mitotic Defects, Inhibits Growth of Melanoma Cells, and Predicts Metastatic Potential of Cutaneous Melanoma. The American Journal of Pathology 166, 1841–1850, doi:10.1016/s0002-9440(10)62493-5 (2005). 15920168 PMC1602405

[59] Brook G. A. . Distribution of B-50(GAP-43) mRNA and protein in the normal adult human spinal cord. Acta Neuropathologica 95, 378–386, doi:10.1007/s004010050814 (1998). 9560016

[60] Sanchez C. G. . Regulation of Ribosome Biogenesis and Protein Synthesis Controls Germline Stem Cell Differentiation. Cell Stem Cell 18, 276–290, doi:10.1016/j.stem.2015.11.004 (2016). 26669894 PMC4744108

[61] Iwata R. . Mitochondria metabolism sets the species-specific tempo of neuronal development. Science 0, eabn4705, doi:doi:10.1126/science.abn4705. 36705539

[62] Warburg O. The chemical constitution of respiration ferment. Science 68, 437–443 (1928). 17782077 10.1126/science.68.1767.437

[63] Warburg O. On the origin of cancer cells. Science 123, 309–314, doi:10.1126/science.123.3191.309 (1956). 13298683

[64] Smith M. A., Parkinso D. R., Cheso B. D. & Friedma M. A. Retinoids in cancer therapy. J Clin Oncol 10, 839–864, doi:10.1200/JCO.1992.10.5.839 (1992). 1569455

[65] Matthay K K. . Long-term results for children with high-risk neuroblastoma treated on a randomized trial of myeloablative therapy followed by 13-cis-retinoic acid: a children’s oncology group study. J Clin Oncol 27, 1007–1013, doi:10.1200/JCO.2007.13.8925 (2009). 19171716 PMC2738615

[66] Axelson H., Fredlund E., Ovenberger M., Landberg G. & Pahlma S. Hypoxia-induced dedifferentiation of tumor cells--a mechanism behind heterogeneity and aggressiveness of solid tumors. Semin Cell Dev Biol 16, 554–563, doi:10.1016/j.semcdb.2005.03.007 (2005). 16144692

[67] Kilburn D. G., Lilly M. D. & Webb F. C. The energetics of mammalian cell growth. Journal of Cell Science 4, 645–654, doi:10.1242/jcs.4.3.645 (1969). 5817088

[68] Yang L. . Serine Catabolism Feeds NADH when Respiration Is Impaired. Cell Metab 31, 809–821 e806, doi:10.1016/j.cmet.2020.02.017 (2020). 32187526 PMC7397714

[69] Abercrombie M. Contact inhibition and malignancy. Nature 281, 259–262, doi:10.1038/281259a0 (1979). 551275

[70] Dong R. . Single-Cell Characterization of Malignant Phenotypes and Developmental Trajectories of Adrenal Neuroblastoma. Cancer Cell 38, 716-+, doi:10.1016/j.ccell.2020.08.014 (2020). 32946775

[71] Bedoya-Reina O. C. . Single-nuclei transcriptomes from human adrenal gland reveal distinct cellular identities of low and high-risk neuroblastoma tumors. Nat Commun 12, doi:ARTN 5309/s41467-021-24870-7 (2021). 10.1038/s41467-021-24870-7 PMC8423786 34493726

[72] Jansky S. . Single-cell transcriptomic analyses provide insights into the developmental origins of neuroblastoma. Nature Genetics 53, 683-+, doi:10.1038/s41588-021-00806-1 (2021). 33767450

[73] Kameneva P. . Single-cell transcriptomics of human embryos identifies multiple sympathoblast lineages with potential implications for neuroblastoma origin. Nature Genetics 53, 694-+, doi:10.1038/s41588-021-00818-x (2021). 33833454 PMC7610777

[74] Kildisiute G. . Tumor to normal single-cell mRNA comparisons reveal a pan-neuroblastoma cancer cell. Science Advances 7, doi:ARTN eabd3311/sciadv.abd3311 (2021). 10.1126/sciadv.abd3311 PMC7864567 33547074

[75] Kastriti M. E. . Schwann cell precursors represent a neural crest-like state with biased multipotency. Embo Journal 41, doi:ARTN e108780 /embj.2021108780 (2022). 10.15252/embj.2021108780 PMC9434083 35815410

[76] Olsen T K. . Joint single-cell genetic and transcriptomic analysis reveal pre-malignant SCP-like subclones in human neuroblastoma. Mol Cancer 23, doi:ARTN 180/s12943-024-02091-y (2024). 10.1186/s12943-024-02091-y PMC11365129 39217332

[77] Hanahan D. Hallmarks of Cancer: New Dimensions. Cancer Discov 12, 31–46, doi:10.1158/2159-8290.CD-21-1059 (2022). 35022204

[78] Yannell K. E., Simmermaker C., Genevieve Van de Bittner & Daniel Cuthbertson. An End-to-End Targeted Metabolomics Workflow. Agilent Application, 5994–5628 (2023).

[79] Tao H., Zhang Y., Zeng X., Shulman G. I. & Jin S. Niclosamide ethanolamine-induced mild mitochondrial uncoupling improves diabetic symptoms in mice. Nat Med 20, 1263–1269, doi:10.1038/nm.3699 (2014). 25282357 PMC4299950

[80] Alasadi A. . Effect of mitochondrial uncouplers niclosamide ethanolamine (NEN) and oxyclozanide on hepatic metastasis of colon cancer. Cell Death Dis 9, 215, doi:10.1038/s41419-017-0092-6 (2018). 29440715 PMC5833462

