# Supplementary material for: Restoring Mitochondrial Quantity and Quality to Reverse Warburg Effect and Drive Tumor Differentiation
Source: Res Sq. 2024 Dec 13:rs.3.rs-5494402. Preprint. [Version 1] doi: 10.21203/rs.3.rs-5494402/v1 (PMC11661309; doi:10.21203/rs.3.rs-5494402/v1)
Supplement: Supplement 1 [file NIHPPRS5494402v1-supplement-1.pdf]

Supplementary Files

This is a list of supplementary files associated with this preprint. Click to download.

- FigureS1.png
- FigureS2.png
- FigureS3.png
- FigureS4.png
- FigureS5.png
- FigureS6.png
- Graphicsabstract.png
- SupplementalVideo1.pptx
